# Immunometabolism at the cornerstone of inflammaging, immunosenescence, and autoimmunity in COVID-19

**DOI:** 10.18632/aging.202422

**Published:** 2020-12-27

**Authors:** Loukman Omarjee, Frédérique Perrot, Olivier Meilhac, Guillaume Mahe, Guilhem Bousquet, Anne Janin

**Affiliations:** 1 Vascular Medicine Department, CHU Rennes, French National Health and Medical Research (Inserm), Clinical Investigation Center (CIC) 1414, University of Rennes 1, Rennes F-35033, France; 2 NuMeCan Institute, Exogenous and Endogenous Stress and Pathological Responses in Hepato-Gastrointestinal Diseases (EXPRES) Team, French National Health and Medical Research (Inserm) U1241, University of Rennes 1, Rennes F-35033, France; 3 Cellular and Molecular Biology Consultant, Rennes F-35033, France; 4 University of Reunion Island, INSERM, UMR 1188 Reunion, Indian Ocean Diabetic Atherothrombosis Therapies (DéTROI), CHU de La Réunion, Saint-Denis de La Réunion F-97400, France; 5 AP-HP Hôpital Avicenne, Oncologie Médicale, Bobigny F-93000, France; 6 Sorbonne University Paris Nord, INSERM, U942, Cardiovascular Markers in Stressed Conditions, MASCOT, Bobigny F-93000, France; 7 Department of Pathology, Paris Diderot University, Sorbonne Paris Cité, Paris F-75010, France

**Keywords:** inflammaging, immunosenescence, immunometabolism, COVID-19, rapamycin-metformin-dimethyl fumarate

## Abstract

Inflammaging constitutes the common factor for comorbidities predisposing to severe COVID-19. Inflammaging leads to T-cell senescence, and immunosenescence is linked to autoimmune manifestations in COVID-19. As in SLE, metabolic dysregulation occurs in T-cells. Targeting this T-cell dysfunction opens the field for new therapeutic strategies to prevent severe COVID-19. Immunometabolism-mediated approaches such as rapamycin, metformin and dimethyl fumarate, may optimize COVID-19 treatment of the elderly and patients at risk for severe disease.

## INTRODUCTION

Chronic low-grade inflammation that develops with aging, termed inflammaging, is a common factor for comorbidities predisposing to severe forms of COVID-19 with acute respiratory distress syndrome (ARDS) [1]. Inflammaging is the long-term result of chronic physiological stimulation of the innate immune system, which can become damaging during aging [2].

Inflammaging leads to T-cell senescence, a T-cell dysfunction state that occurs in chronic infections [3–5]. Reduced counts with functional exhaustion of T-cells and cytokine release syndrome have been identified as adverse factors in patients suffering from SARS-CoV-2 infection [3, 4]. Severe COVID-19 can therefore mimic a state of immune senescence [6]. Immunosenescence is defined as age-related alteration to the immune system leading to a progressive reduction in ability to trigger effective antibody and cellular responses to infections and vaccinations [7].

Immunosenescence is linked to autoimmunity in age-related disorders [8]. It can be potentiated by COVID-19, thus highlighting the role of infectious agents in triggering an autoimmune response [9].

Investigation into possible factors for inflammaging, immunosenescence and autoimmunity in COVID-19 patients is ongoing. Immunometabolism dysfunction may be at their core. Immunometabolism represents the changes that occur in intracellular metabolic pathways within immune cells during activation [10, 11]. It is widely accepted that T-cell senescence is due in part to dysfunction in normal glycolytic CD8 T-cell metabolism [10]. Metabolic pathways are important regulators of immune differentiation and activation, and as such influence the immune response to SARS-CoV-2 [11, 12].

This review details how SARS-CoV-2 might induce or amplify inflammaging, immunosenescence and autoimmunity from an immunometabolic perspective and puts forward therapeutic approaches for restoring T-cell functionality.

## Severe forms of COVID-19 promoted/enhanced by inflammaging and immunosenescence

Risk factors for severe COVID-19 have been characterized by China Center for Disease Control and Prevention findings based on 44 672 COVID-19 patients [13]. Old age and age-related disorders such as cardiovascular disease, obesity, diabetes mellitus, chronic respiratory disease, hypertension, and cancer were linked to greater risk of death [13, 14]. A meta-analysis of seven studies involving 1576 COVID-19 patients indicated that the most prevalent comorbidities were hypertension (21.1%, 95% CI: 13.0–27.2%) and diabetes mellitus (9.7%, 95% CI: 7.2–12.2%), followed by cardiovascular (8.4%, 95% CI: 3.8–13.8%) and respiratory disease (1.5%, 95% CI: 0.9–2.1%) [15]. In addition, of the 3615 individuals who tested positive for COVID-19, 775 (21%) were obese, and 595 (16% of the total cohort) were severely obese [16]. In 124 consecutive COVID-19 patients admitted to the ICU, 47.6% were obese and 28.2% severely obese [17].

Biologically, onset of severe COVID-19 is characterized by a cytokine storm with hyper secretion of pro-inflammatory cytokines [18].

Age-related disorders such as risk factors for COVID-19 are connected to low-grade, persistent inflammation (inflammaging) and have detrimental effects on the immune system [19].

**Figure 1:**
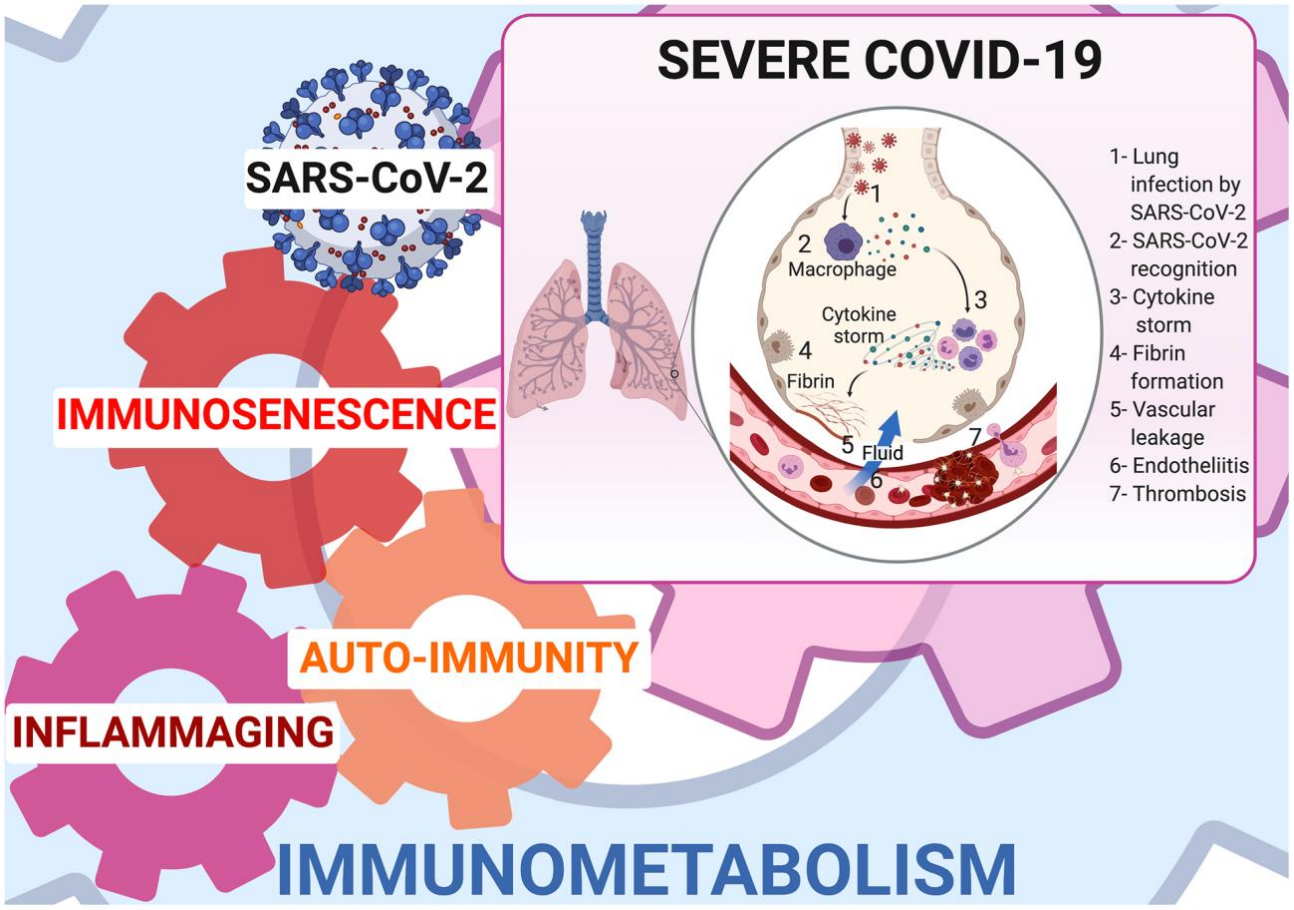
**Immunometabolism at cornerstone of inflammaging, immunosenescence, and autoimmunity in COVID-19.**

Inflammaging is linked to mitochondrial dysfunction [14], SASP and age-related autoimmune predisposition [20]. Mitochondrial dysfunction has been shown in SARS-COV-2 infected lung cell lines where upregulation of genes involved in mitochondrial cytokine/inflammatory signalling and downregulation of genes involved in organization, respiration and autophagy has been demonstrated [14]. SASP releases cytokines (IL-1α/β, IL-6, IL-8, TGF-β, and TNF-α) [21], some of which are able to increase senescence via a “bystander effect” [22]. IL-1α is the main upstream regulator of SASP, while IL-1β and TGF-β are senescence transmission mediators, and IL-6 and IL-8 reinforce autocrine senescence [22]. In the elderly, especially elderly men, IL-6 is chronically upregulated [23] and its elevation is predictive of mortality [24]. Tissue accumulation of senescent cells and SASP secretion are instrumental in provoking a cytokine storm, that is a major contributor to ARDS and multiple organ dysfunction syndrome [18, 19]. (Figure 1)

## COVID-19: an immunosenescence model

### Cellular senescence

Senescence is a biological process that implicates each body cell [21, 25]. The concept of senescence originates from a work by Hayflick et al., describing loss of replicative capacity in fibroblast cell cultures [26]. Aging cells cease to function properly, fail to accomplish their normal tasks, and lose their ability to divide [21, 25]. Instead of dying off, they accumulate in tissues [21, 25]. Cellular senescence is thus a major cause of aging and age-related diseases, including diabetes mellitus, obesity, and cardiovascular disorders [21, 25].

Senescent cells also prevent surrounding healthy cells and tissues from functioning at peak capacity [27], and they secrete harmful compounds such as SASP [21, 25]. SASP induces senescence in other cells, and so the destructive process continues [21, 25]. Senescent cells are associated with dysfunctional mitochondria [14], contribute to chronic inflammation [21, 25] and promote clotting [28] and clotting-related conditions such as sepsis-induced coagulopathy or disseminated intravascular coagulopathy that are found in COVID-19 [29].

### Immunosenescence in COVID-19

Senescence also affects immune cells, and features of immunosenescence have been found in severe COVID-19 patients [3, 4, 30–33].

Immunosenescence is characterized by decreased naïve T-cells, increased memory T-cells, and poor response to newly-encountered antigens and vaccines [34, 35]. T-cells play a vital role in viral clearance through cytotoxic molecule secretion [4].

Higher ongoing SARS-CoV-2 viral loads cause overstimulation of CD8 T-cells via TCR signaling, inducing CD8+ T-cell senescence [36, 37]. In severe COVID-19, circulating T-cells show signs of senescence by expressing PD-1, Tim-3, CTLA-4 and TIGIT [3, 4, 30, 31]. CD4+ and CD8+ T-cells have been observed in patients over 60 years of age and those receiving ICU care. There is an inverse correlation between decrease in total number of T-cells and patient survival [3]. Moreover, senescent CD8+ T-cells are unable to secrete cytotoxic perforin, granzyme and IFN-γ [4] in these patients.

Senescent T-cells also secrete the cytokines, chemokines, proteases and growth factors [38] that define SASP [38], and contribute to the cytokine storm that occurs in severe COVID-19 [3].

Pre-existing factors that render patients vulnerable to COVID-19 could lead to immunosenescence [20, 39].

Firstly, CMV infection is highly prevalent (>90%) in the elderly, and leads to immunosenescence through phenotypic changes and loss of T-cell repertoire diversity [40]. Higher CMV viral loads cause CD8+ T-cells to overstimulate via TCR signaling, thus inducing CD8+ T-cell senescence [37]. Diversity in clonally-expanded CMV-specific memory CD8+ T-cells and concomitant decreased naïve T-cells results in poor response to influenza vaccines in the elderly [7]. Chronic CMV infection may bring about a diminished immune response to SARS-CoV-2 infection and to any future vaccination [41].

Secondly, aging attenuates the upregulation of co-stimulatory molecules critical to T-cell priming and reduces antiviral IFN production by means of alveolar macrophages and dendritic cells [42]. The ability of DCs and macrophages to elicit CD8+T-cell response and proliferation and to release antiviral cytokines is thus impaired [42]. In the lung, delayed IFN production leads to cellular damage to airway and alveolar epithelia, and contributes to cytokine storm [43]. In 50 COVID-19 patients, severe and critically ill patients had impaired type I IFN response, persistent blood viral load and exacerbated inflammatory response [44]. The international “COVID Human Genetic Effort” consortium has shown that inborn errors of TLR3- and IRF7-dependent type I IFN immunity can underlie life-threatening COVID-19 pneumonia in 3%-4% of patients with no prior severe infection [45].

Autoimmune diseases can also neutralize type I IFN activity via IgG auto-antibodies at the onset of critical disease as found in 10% of severe forms of COVID-19 [45, 46].

Coronaviruses themselves can hinder early induction of type I IFN [43] through proteases that antagonize STING whose role is to recognize cytosolic viral DNA [43], or in SARS-CoV-1 through addition of a 2’ O-methyl group to viral RNA, to evade detection by MDA-5 [43].

Taken together, elderly patients and those with risk factors for severe COVID-19 cannot mount an efficient adaptive antiviral immune response [43].

Thirdly, age-related thymic involution reduces the output of naïve T-cell and TCR repertoire [40], thus producing a characteristic immunosenescence profile [40]. Lymph nodes undergo age-related changes, and become less able to maintain naïve T-cell homeostasis and to coordinate new immune responses to emerging infections [43]. The balance of bone marrow immune cell production is affected, involving reduced lymphopoiesis and enhanced myelopoiesis [47]. Senescent lymphoid organs can compromise an efficient immune response against COVID-19.

Fourthly, angiotensin converting enzyme-2 (ACE2) expression which is the primary target receptor for SARS-CoV-2 occurs in the lungs, oral mucosa, gut enterocytes, and endothelial cells [42]. It has a protective effect on endothelial cells and lung function by limiting angiotensin II-mediated pulmonary capillary leakage and inflammation [42]. ACE2 downregulation by the spike protein of SARS-CoV-2 in the lung might be implicated in ARDS through release of proinflammatory chemokines and cytokines [42].

Fifthly, obesity, metabolic syndrome, and diabetes mellitus are linked to systemic immunometabolic inflammation. In this “metaflammation” the circulating cytokine levels are increased by activation of the NLR family pyrin domain-containing 3 (NLRP3) inflammasome/IL-1 axis, NF-κB and JNK pathways [14, 48, 49]. Interaction between macrophages and adipocytes are early molecular events in subclinical inflammation, further inducing insulin resistance, glucotoxicity, lipotoxicity, endothelial dysfunction, systemic inflammation and cardiovascular disease [48]. Obesity is linked to dysregulated adaptive immunity and failure to generate antibodies following infection or vaccination [49]. Obesity entails an increased risk for hypertension, diabetes mellitus, and cardiovascular disease, three of the most important underlying conditions in severe COVID-19 [50].

Lastly, telomere shortening, that is linked to cellular aging, may play a role in severe COVID-19 [42]. Older adults whose telomeres were comparatively short had mortality rates over eight times higher from infectious disease and over three times higher from heart disease than those whose telomeres were longer [51]. T-cells from chronically virus-infected individuals age prematurely or are senescent due to telomere attrition and erosion [7]. Accelerated telomere loss in various leukocyte subpopulations is a common feature of ARDS and autoimmune disease and enhances proinflammatory cytokine production [42]. The mechanisms linking telomere attrition, cell senescence, and aging are not restricted to the inhibition of cell division, but also rely on the acquisition of pro-inflammatory secretome by senescent cells known as SASP [42, 52]. Prompt recovery of the immune response requires massive lymphopoiesis which is telomere length-dependent [53], and in COVID-19 lymphopenia is linked to mortality [32].

A vicious circle is then established whereby pre-existing senescence factors worsen acquired senescence, which itself amplifies age-related senescence.

## Immunosenescence and autoimmunity in COVID-19

Following infection by COVID-19, there have been reports [9] of autoimmune disease onset including autoimmune thrombocytopenia [54], autoimmune hemolytic anemia [55], cold agglutinin disease [56], Guillain-Barré syndrome [57], encephalopathy involving choreiform movements [58], and antiphospholipid syndrome [59].

Lymphopenia is one of the hallmarks of severe COVID-19 [60]. It is also a characteristic feature of human SLE and RA [8], and a risk factor for NOD mouse autoimmune diabetes [8]. Lymphopenia is linked to premature aging of the immune system [8]. When lymphopenia occurs, the CD4+ T-cells undergo homeostatic proliferation, which, when sustained, increases the risk for autoimmunity [61]. SARS-CoV-2 may act as a triggering factor for activation of autoreactive lymphocytes [9].

In COVID-19, tissue infection can also induce a local innate immune response that involves overexpression of costimulatory molecules and cytokines by tissue antigen-presenting cells [62]. These cells are able to stimulate self-reactive T-lymphocytes [63]. COVID-19 thus has the capacity to disrupt T-cell tolerance and promote the survival and activation of self-reactive T-cells [9]. Thymic epithelial cell involution, which plays an essential role in central T-cell self-tolerance, also contributes to self-tolerance breakdown [61].

TCR diversity deteriorates with age, and oligoclonal cell populations are common in the elderly [64]. This population also exhibits a diminished response to vaccination indicative of a decline in T-cell functional response, and a Th2 profile [64]. This switch from a Th1 to Th2 profile that is also observed in severe COVID-19 patients may lead to an ineffective antiviral immune response [3, 6, 65].

Making use of IL-6 [66], COVID-19 expands Th17 cells [30, 67] which provide antibacterial protection, but are also associated with the development of autoimmune diseases [68]. Pro-inflammatory Th17 cells are in homeostatic balance with anti-inflammatory Tregs [30, 67]. Tregs are able to suppress autoimmune phenomena and dampen allergic reactions, but they can also inhibit a protective immune response to invading pathogens [30, 35]. The Th17/Treg ratio is higher in patients with SLE, RA, multiple sclerosis, and inflammatory bowel disease much the same as in severe COVID-19 [30, 68]. Follow-up of COVID-19 patients is important to assess the occurrence rate of induced autoimmune diseases.

Since SARS-CoV-2 is able to produce antigens that resemble self-antigens, immune responses to these peptides can result in an autoimmune attack by molecular mimicry [9]. This may account for the autoimmune neurological complications and autoimmune anemia observed in COVID-19 patients [69, 70].

In the brain, damage to the respiratory pacemaker known as the pre-Bötzinger complex (preBötC) through mimicry between viral and neuronal proteins of the preBötC contributes to respiratory failure in COVID-19 [69]. In autoimmune hemolytic anemia, the erythrocyte membrane protein ANK-1 shares an epitope that is 100% identical to the SARS-CoV-2 spike surface glycoprotein [70]. Age-related conditions associated with severe COVID-19 also provoke endotheliitis and endothelial dysfunction [29]. Hence abnormal expression of plasma membrane molecules in endothelial cells, resulting from post-translational modification of intracellular proteins such as heat shock proteins, can predispose cells and tissues to molecular mimicry leading to autoimmunity [71].

## COVID-19: a systemic disease mimicking systemic lupus erythematous crosstalk between immunosenescence, autoimmunity and immunometabolism

The metabolism drives immune T-cell activation and differentiation [10, 11, 72]. Cytokines, metabolic substrates, epigenetic reprogramming and other microenvironmental factors regulate this process by influencing T-cell activation and function [10, 11, 35, 72]. The metabolic status of T-cells depends on their differentiation stage [10]. Both naïve CD4+ and CD8+ T-cells use the oxidative phosphorylation pathway at rest [10]. Treg and memory CD4+ T-cells use fatty acid oxidation to support oxidative phosphorylation and cell proliferation [10]. Differentiated effector CD4+ cells, such as Th1 and Th17, prefer glutaminolysis, rapid glycolysis and fatty acid synthesis [10]. Upon activation, the naïve T-cell metabolism shifts towards aerobic glycolysis (Warburg effect) and embarks on a pentose phosphate pathway to generate nucleotides, amino acids, lipids and NADPH so as to enhance cellular antioxidants [10].

SLE is a chronic autoimmune disease characterized by abnormal T-cell responses to self-antigens resulting in multi-organ involvement [73]. Lymphopenia and senescent T-cells, two characteristic features of SLE, are also found in severe COVID-19 patients. In SLE, senescent PD-1+ CD4+ and PD-1+ CD8+ T-cells are correlated with increased disease activity and autoantibody production [74, 75].

A link has been made between immunosenescence in SLE patients and immunometabolic alterations such as mitochondrial dysfunction, oxidative stress, glycolysis, glutaminolysis and lipid metabolism contributing to pro-inflammatory T-cell responses [73]. In SLE, T-cells have: i) dysfunctional oxidative phosphorylation pathways which can reduce Treg counts and functional exhaustion; ii) enhanced glycolysis which increases Th17-associated autoimmunity; iii) reduced naïve CD4+T-cells and increased memory CD4+T-cells [76].

In CD4+ T cells from SLE patients, autophagy suppression induced by mTOR activation leads to their dysfunction in the differentiation and effector functions [77].

The energy metabolism of senescent PD-1+ CD4+ T-cells is different from non-senescent CD4+ T-cells [75]. When T-cells fail to use aerobic glycolysis, they enhance PD1 expression and develop a defect in IFN-γ production [14, 78]. The same metabolic mechanism could account for INF- γ deficiency in severe COVID-19 patients [11].

As in SLE, lymphocyte subsets with higher central memory CD4+T-cell subpopulations (CD45RA+) were found in 39 severe COVID-19 patients [30]. CD45RA expression characterizes senescent T-cell populations which sustain low proliferative activity, high levels of DNA damage and loss of telomerase activity [79].

These 39 severe COVID-19 patients were also characterized by higher percentages of CD8+ T-cell terminal effector cells expressing CD38 alone or in combination with CD57, and by activated effector memory cells expressing PD1 or CD57 [30]. They displayed significantly lower percentages of naïve and central memory T-cells, which could suggest that the CD8+ T-cell compartment was senescent in these patients. [30] In SLE patients suffering from infections, the CD8+CD38^high^T-cell population is expanded [80]. These cells demonstrate reduced cytotoxic function [80] similar to senescent T-cells found in severe COVID-19 [3, 4, 30, 31].

## Targeting immunosenescence and immunometabolism to prevent cytokine storm in COVID-19

**Figure 2:**
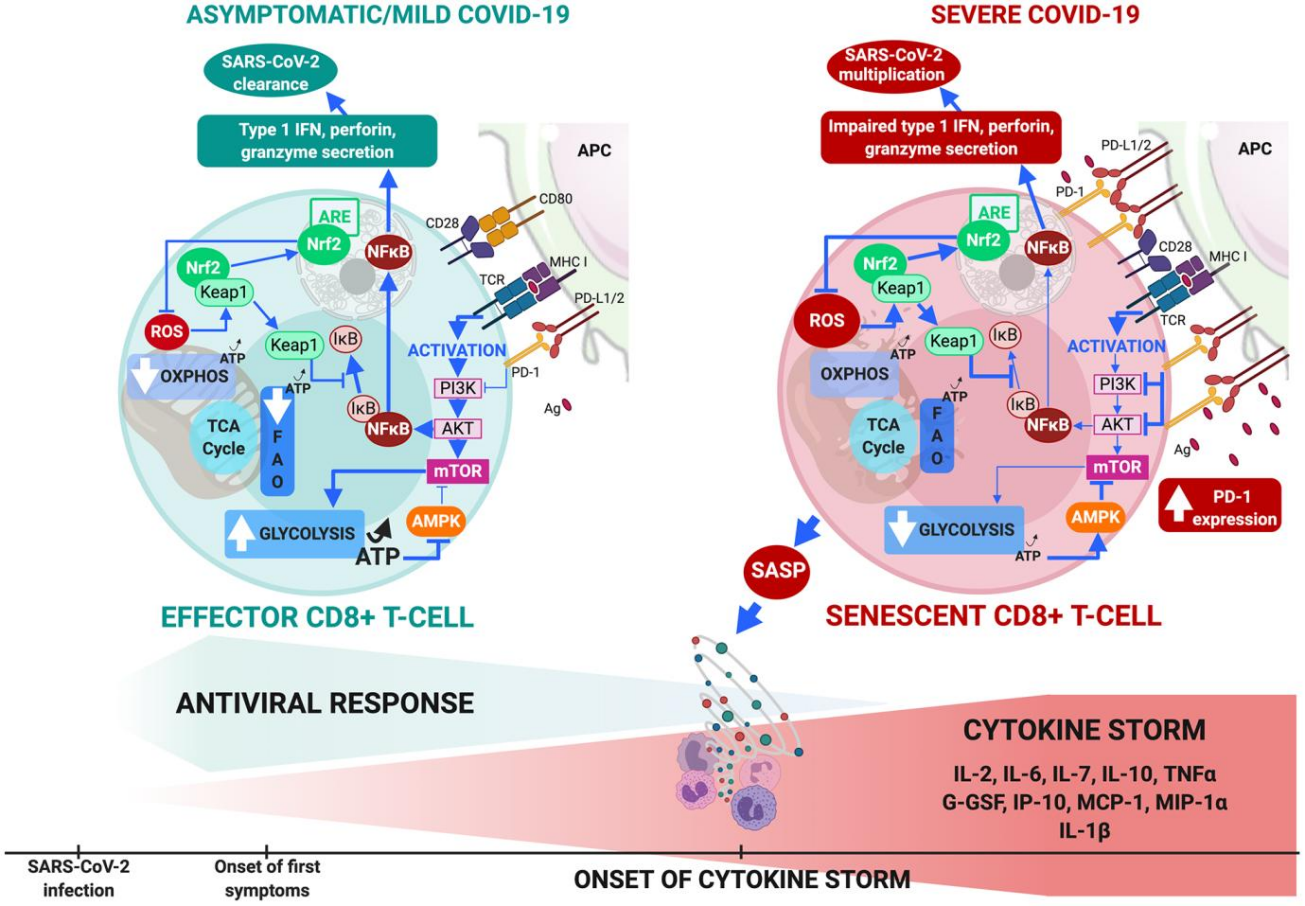
**CD8+ T-cell metabolism in COVID-19.** (**A**) In comorbidity-free patients developing asymptomatic/mild symptomatic forms of COVID-19, cytotoxic immune response mediated by effector CD8+ T-cells results in eradication of virus and patient recovery. Normal antigen levels in antigen-presenting cells and cytokine levels (interleukin IL-2 released by helper CD4+ T-cells) stimulate TCRs and co-receptors such as CD28, thus enhancing mTOR signaling via PI3K and protein kinase B that increases glycolysis. Cells shift from OXPHOS/FAO to glycolytic-based metabolism, whereby anabolic processes activate effector cells to clear infection. This includes production of cytotoxic factors (type 1 Interferon, granzyme, perforin) and enhanced proliferation. Massive increase in glycolysis results in production of ATP (less than OXPHOS but sufficient to inhibit AMPK, preventing mTOR pathway blockade. ROS production activates Nrf2, reducing inflammation and apoptosis by inhibiting NF-κB and pro-inflammatory cytokine production. (**B**) Aging and age-related disorders cause CD8+ T-cell senescence in severe COVID-19. Excess antigens upregulate inhibitory receptors (programmed death-1: PD-1) that block TCR activation, thus reducing signaling required for glycolytic metabolic phenotype itself crucial to proper effector functioning. Malfunction is compounded by upregulation of PD-1 expression-enhancing transcription factors, reduction in helper cell survival and proliferation signaling (IL-2), and increase in inhibitory signals. Senescent CD8+ T cells secrete SASP, paracrinely amplifying production of inflammatory cytokines and triggering cytokine storm. Massive decrease in glycolysis causes fall in ATP production and fails to sufficiently inhibit AMPK which then partially inhibits mTOR pathway. Substantial ROS production activates Nrf2 but fails to inhibit NF-κB pathway and pro-inflammatory cytokine production. These events combined make cells malfunction metabolically, inhibit cytotoxic function and exhaust the phenotype.

Three therapeutic approaches that target T-cell senescence by reversing metabolic dysfunction are available in severe COVID-19 (Figure 2).

### Rapamycin, an mTOR inhibitor

The PI3K/Akt/mechanistic mammalian target of the rapamycin (mTOR) pathway is a central regulator of inflammation within the immune system, acting as a sensor for oxidative stress and cell metabolism [6, 11, 12]. mTOR pathway activation increases protein synthesis, glycolysis and other proliferation and survival processes [12].

In COVID-19, the mTOR pathway may provide valuable targets for controlling cell injury, oxidative stress, impaired autophagy and onset of hyperinflammation [6, 77]. mTORC1 mediates Th1 and Th17 differentiation upon viral antigen presentation by dendritic cells (DC) and mTORC2 mediates Th2 differentiation. Both complexes restrict Treg differentiation [81].

In T-cell metabolism, the oxidative stress and inflammation that activate mTORC1 can be blocked by N-acetylcysteine and/or rapamycin (sirolimus) in SLE patients [82]. Rapamycin, an mTOR inhibitor with the capacity to promote autophagy and suppress SASP, may restore T-cell functionality and attenuate cytokine storm in COVID-19 [6, 77]. In elderly patients with increased senescent PD-1+ T-cells, everolimus (an analog of rapamycin) enhanced immune function, and improved T-cell responses to antigenic stimulation with an acceptable risk/benefit balance [5]. In elderly coronary artery disease patients, rapamycin brought down serum senescence markers through IL-6 suppression [23]. In patients infected with the H1N1 influenza virus, early adjuvant rapamycin therapy over a short period (2 mg/day for 14 days) was significantly linked to enhanced viral clearance, greater improvement in lung injury (i.e. less hypoxemia), and a decrease in multiple organ dysfunction [83]. Duration of mechanical ventilation in survivors was also shorter [83]. In mouse models, H1N1 causes acute lung injury via an IL-17-dependent mechanism [84]. mTOR blockade by rapamycin may inhibit Th17 cell expansion in COVID-19 patients similarly to SLE patients [85]. H1N1 and SARS-CoV-2 both activate mTOR, and NLRP3 inflammasome pathways [86, 87] leading to production of IL-1β, a mediator of lung inflammation, fever and fibrosis. The NLRP3 pathway induces pyroptosis, that is a hyperinflammatory form of cell death [88]. Rapamycin inhibits H1N1-induced mTOR pathway activation, and thus IL-1β secretion [6]. In COVID-19 the binding of SARS-CoV-2 to TLR, which induces IL-1β production, is potentially reversed by rapamycin [89]. Rapamycin promotes de novo Foxp3 expression in naïve T-cells, leading to Treg proliferation and survival [81]. Rapamycin inhibits effector T-cell proliferation and enhances Treg accumulation [81].

In addition, rapamycin was recently identified in a network-based drug repurposing study as a candidate for potential use in COVID-19 [89]. When administered early in the onset of the cytokine storm phase, it is possible that rapamycin prevents progression to severe forms of COVID-19 through the down-regulation of SASPs, of the mTOR-NLRP3-IL-1β axis, of the IL-6 pathway, and of senescent T-cell counts [6].

**Figure 3:**
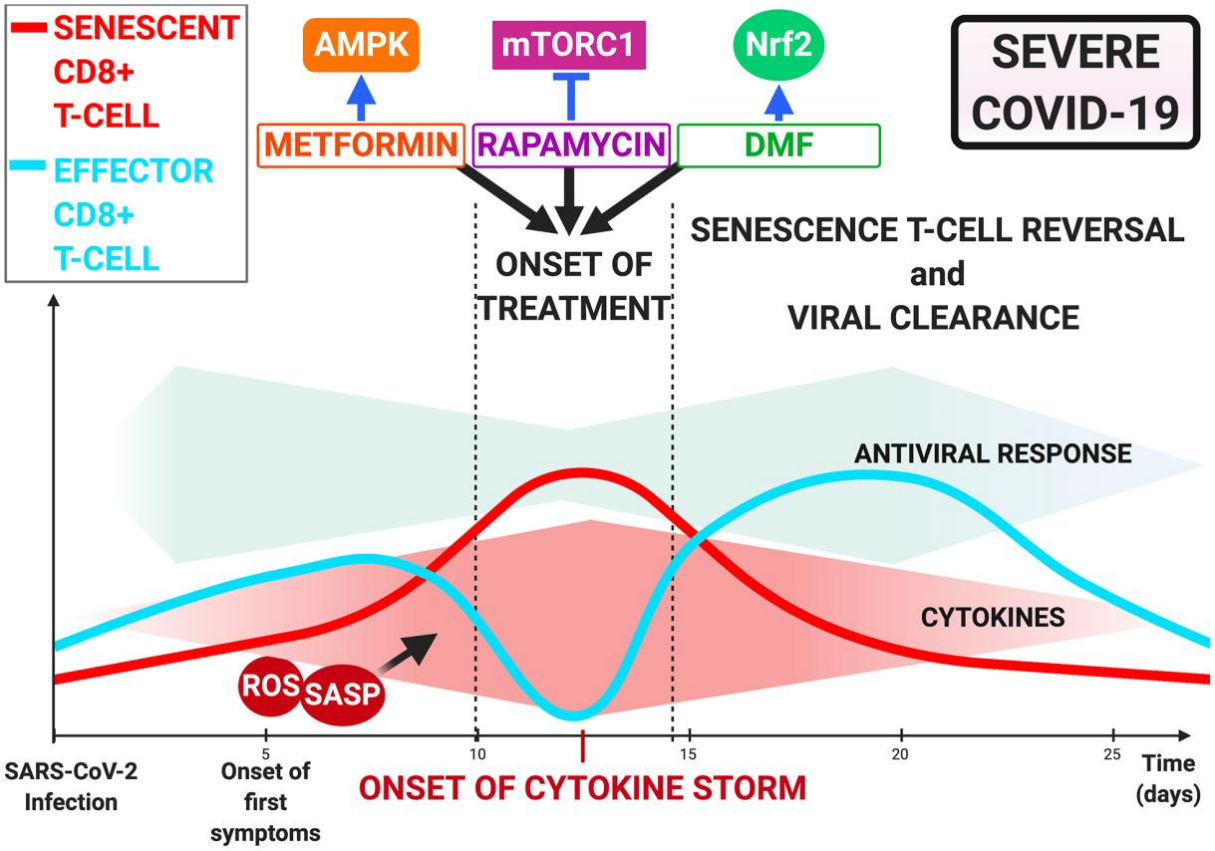
**Immunometabolism-Mediated therapies targeting T-cell dysfunction in COVID-19.** Onset of cytokine storm as treatment opportunity via rapamycin, metformin, and dimethyl fumarate. Inhibition of mTOR by rapamycin, AMPK by metformin and Nrf2 activation by dimethyl fumarate may restore CD8+ T-cell functionality and improve antiviral response and patient outcome.

In combination with antiviral therapy, rapamycin is likely to optimize treatment of COVID-19 patients with advanced chronological age, and/or co- morbidities, or those with reduced T-cell counts who are more likely to progress to severe disease [6] (Figure 3).

### Metformin: an AMPK activator

AMPK regulates tissue energy metabolism and controls immune responses through its cooperation with immune signaling pathways [90]. AMPK is also able to positively regulate autophagy [77]. AMPK signaling inhibits pro-inflammatory NF-κB pathways [90]. AMPK also downregulates JAK/STAT signaling pathways, known to drive cytokine signaling, cell growth, and apoptosis [90].

AMPK increases expression of and stabilizes ACE2 via phosphorylating ACE2 Ser^680^ in HUVECs [91]. T-cell activation in response to TCR engagement is linked to robust AMPK activation via a Ca2+/calmodulin-dependent protein kinase pathway, an ubiquitous and evolutionarily conserved pathway that regulates energy homeostasis [92].

Re-purposing metformin, which activates AMPK in hepatocytes, may be useful in COVID-19. While metformin was originally introduced as an anti-influenza drug, it has glucose-lowering side effects [91] and is therefore a first-line therapy for diabetes mellitus [93]. It can also reduce inflammation and obesity [93] and is currently under evaluation for aging [94] and SLE [95]. Metformin lessens the hallmarks of aging by improving nutrient sensing, enhancing autophagy and intercellular communication, protecting against macromolecular damage, delaying stem cell aging, modulating mitochondrial function, regulating transcription, and lowering telomere attrition and senescence [96]. Mechanistic studies have illustrated its role in Th17 cell differentiation via the AMPK/mTOR/STAT3 pathway [95]. In COVID-19, as in SLE, an increase in Th17 cells occurs^38^. Metformin can also normalize CD4+ T-cell glucose metabolism via inhibition of mitochondrial complex I and oxidative phosphorylation [95]. Since glucose metabolism is crucial to the activation, proliferation and differentiation of CD4+ T-cells, metformin can reduce overactive effector T-cells (including Th1 and Th17) and proinflammatory cytokines (including interferon IFN-γ and IL-17) in SLE [95]. CD8+ T-cells play a vital role in viral clearance, particularly through secretion of cytotoxic molecules such as perforin, granzyme and IFN-γ [6]. At doses administered in the treatment of diabetes mellitus, metformin may restore T-cell functionality and attenuate the cytokine storm in COVID-19 [90, 91, 97].

Furthermore, metformin results in phosphorylation of ACE2 by virtue of AMPK activation, and mitigates binding with SARS-CoV-2 [91].

Clinical reports suggest that treating diabetes mellitus patients with metformin decreases the risk of death in COVID-19 [97–99], possibly due to the effect of TNF-α [98]. In an observational study involving 2333 hospitalized COVID-19 patients, metformin was significantly associated with reduced mortality in obese or diabetic patients [98]. In 600 patients with diabetes mellitus and COVID-19, administration of metformin was associated with a reduction in mortality of almost 70% after adjustment for multiple confounders [99]. In four other studies, metformin was associated with an overall reduction in death of 25% (P < .00001), albeit with relatively high heterogeneity (I² = 61%) [97].

Since mTOR is a downstream signaling molecule in the AMPK pathway [90], metformin combined with rapamycin may offer the possibility of restoring T-cell functionality and preventing severe progression in COVID-19, provided it is initiated early in the cytokine storm phase (Figure 3).

### Dimethyl fumarate: a Nrf2 activator

Nrf2 is a redox-sensitive transcription factor that regulates the expression of ARE-dependent genes, among which HO-1 and NQO1 [100]. Under normal conditions, Nrf2 is retained in the cytoplasm in a silent form by its repressor protein, Keap1, which contains reactive cysteine residues. Oxidative stress modifies Keap1 cysteine residues, enabling Nrf2 translocation to the nucleus where it binds to ARE [100].

Due to its antioxidative stress activity, Nrf2 plays a protective role in antiviral and antibacterial processes in lung infections [101]. In COVID-19 pneumonia, Nrf2 activators may i) inhibit virus entry by inducing antioxidant enzyme gene expression; ii) protect lung alveoli through induction of antiviral IFN, RIG-I and IFN-β gene expression; iii) have anti-inflammatory and anti-apoptotic effects by inhibiting NF-κB, TNF-α, IL-6, MCP-1, MIP-2 and downregulating selectins and VCAM-1; iv) inhibit TLR expression of these receptors [101].

Re-purposing DMF, which is a drug that has been approved for the treatment of multiple sclerosis (MS), may therefore be of use in COVID-19 [101]. DMF suppresses inflammation through Nrf2 activation, NF-κB blockade and glutathione modulation [101]. It can inhibit SARS-CoV-2 entry into lung alveolar cells via: i) an increase in anti-protease secretory leukocyte protease inhibitor; a decrease in transmembrane serine protease; iii) ACE2 upregulation that competes with the virus at the binding site [101]. To date, there have been no reported cases of severe COVID-19 infections in DMF-treated MS patients [102]. DMF treatment was not discontinued in young and non-lymphopenic MS patients affected by COVID-19 [102, 103].

For COVID-19 patients, further assessment of the benefits of administering DMF at an early stage to prevent a cytokine storm from occurring is required (Figure 3).

## CONCLUSIONS

The COVID-19 pandemic has demonstrated that this disease follows the classic kinetics of a viral infection in young subjects with no comorbidities. In the elderly and in patients with co-morbidities, the adaptive immune response is less efficient due to senescent lymphocytes and inflammaging. A link can be established between senescence and autoimmune manifestations induced by COVID-19. As in SLE, T-cell metabolism becomes deregulated. Therefore, there is a strong possibility that rapamycin, metformin and DMF, as an immunometabolism-mediated approach, optimize COVID-19 treatment in the elderly and in patients who have risk factors for severe disease.

## Reprint Permission

The authors do hereby declare that all illustrations and figures in the manuscript are entirely original and do not require reprint permission.
